# Go West: A One Way Stepping-Stone Dispersion Model for the Cavefish *Lucifuga dentata* in Western Cuba

**DOI:** 10.1371/journal.pone.0153545

**Published:** 2016-04-15

**Authors:** Damir Hernández, Didier Casane, Pedro Chevalier-Monteagudo, Louis Bernatchez, Erik García-Machado

**Affiliations:** 1 Centro de Investigaciones Marinas, Universidad de La Habana, Calle 16, No. 114 entre 1ra y 3ra, Miramar, Playa, La Habana, 11300, Cuba; 2 Laboratoire Evolution, Génomes, Comportement, Ecologie, CNRS, Université Paris-Sud, UMR 9191, IRD UMR 247, Gif-sur-Yvette, France; 3 Université Paris-Diderot, Sorbonne Paris-Cité, Paris, France; 4 Acuario Nacional de Cuba, Calle 60, Miramar, Playa, La Habana, 11300, Cuba; 5 Institut de Biologie Intégrative et des Systèmes (IBIS), Pavillon Charles-Eugène Marchand, Université Laval, Québec, QC, G1V 0A6, Canada; CNRS, UMR 9197, FRANCE

## Abstract

Consistent with the limited dispersal capacity of most troglobitic animals, almost all *Lucifuga* cavefish species have very narrow geographic distribution in Cuba. However, one species, *L*. *dentata*, has a wide but disjointed distribution over 300 km in the west of the island. In order to estimate the relative role of vicariance and dispersal in the unexpected *L*. *dentata* distribution, we obtained partial sequences of the mitochondrial DNA (mtDNA) cytochrome b (*cyt*b) gene and control region (CR), and then applied Approximate Bayesian Computation (ABC), based on the identification of five genetic and geographic congruent groups of populations. The process that best explains the distribution of genetic diversity in this species is sequential range expansion from east Matanzas to the western Pinar del Río provinces, followed by isolation of groups of populations. We found relative high haplotype diversity and low nucleotide diversity in all but the Havana group, which has high values for both diversity parameters, suggesting that this group has been demographically stable over time. For two groups of populations (Cayuco and Bolondrón), the mismatch distribution analyses suggests past demographic expansion. In the case of the Cayuco region, the star like relationships of haplotypes in the network suggests a recent founding event, congruent with other evidence indicating that this is the most recently colonized region. Over all, the results suggest that a combination of habitat availability, temporal interconnections, and possibly the biological properties of this species, may have enabled its dispersal and range expansion compared to other species of the genus, which are more geographically restricted.

## Introduction

Some general patterns have emerged from studies on genetic polymorphism in obligate subterranean aquatic species (stygobionts). These patterns are: relatively low genetic diversity, reduced if any gene flow between geographicaly close populations, and few dispersal events over long distances [1, 2, 3, 4, 5, 6, 7, 8, 9, 10]. These observations have lead to the prevailing model of groundwater populations as being characterized by high intra-population genetic homogeneity and high inter-population genetic differentiation that could eventually lead to speciation [11, 12, 13, 14, 15]. However, some cases of relatively wide range distributions and/or high genetic diversity and/or extensive gene flow have also been reported in stygobionts [7, 15, 16, 17, 18].

The blind cave dwelling fish *Lucifuga dentata* is a stygobiont (Poey 1858) [19]. The most recent study on the distribution and genetic differentiation of the Cuban *Lucifuga* species revealed several cryptic lineages restricted to narrow karst patches, with the sole exception of *L*. *dentata* sensu stricto, which has a fragmented distribution over 300 km in Cuban south-western karst areas [10]. A phylogeographic analysis based on data from partial sequences of *cyt*b gene revealed three to four highly isolated geographic groups of populations and showed a significant association between haplotype clades and their geographical distribution. A Nested Cladistic Phylogeographic Analysis (NCPA) suggested long-distance colonization and fragmentation as mechanisms shaping these patterns. Other evidence (*i*.*e*. overlapping distributions of divergent evolutionary lineages, the predominance of unique mtDNA haplotypes at most of the caves and closely related lineages in distant karst patches), together with the highly fragmented nature of underground cave systems, strongly supports dispersal followed by allopatric divergence as the major mechanism promoting speciation in *Lucifuga*. Here we test these two hypotheses, dispersal versus fragmentation at different scales, in *L*. *dentata* by Approximate Bayesian Computation (ABC). We increased the sample size for some regions and added the mtDNA control region sequence to the *cyt*b data set previously used [10].

## Materials and Methods

### Population sampling

**Fig 1:**
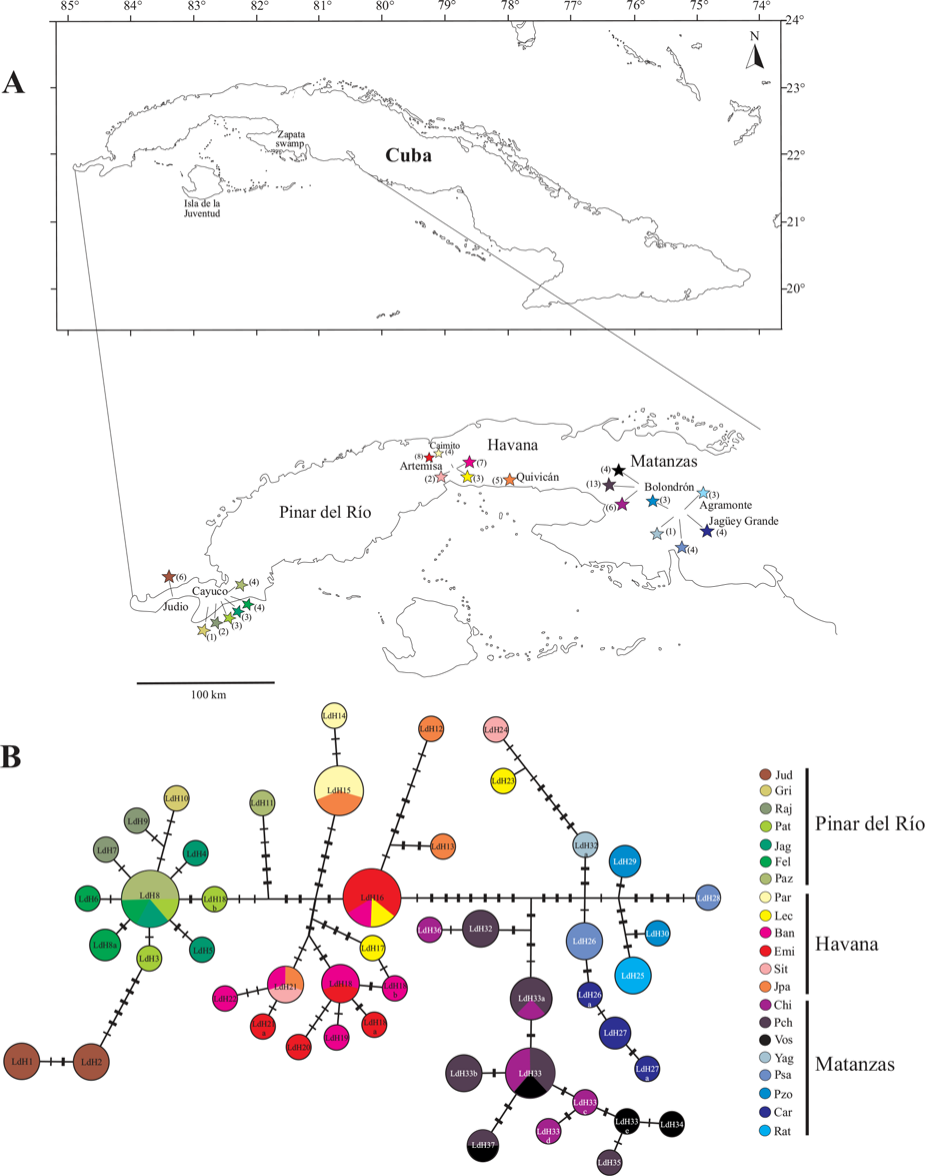
A) *Lucifuga dentata* sampling localities. Sample sizes for each locality are shown within the parentheses. B) Network constructed using the combined *cyt*b and CR sequences. Crossbars denote mutational steps (thinner lines for *cyt*b and thicker lines for CR). Haplotypes IDs with an extra final lower case letter are additional haplotypes identified using combined *cyt*b and CR region sequences compared to the previous study using *cyt*b sequences only [10]. In both (A and B) colours represent a particular cave. Cave name abbreviations are shown in Table 1.

A total of 90 *Lucifuga dentata* sensu stricto individuals (76 of the 80 previously analyzed by García-Machado et al. [10] and 14 newly sampled from two localities) from 21 localities, representing most of the species distribution, were examined (Fig 1, Table 1). Individuals were collected, using hand nets, under field access permission number LH 112 AN (135) 2013, from the Cuban Center for Environmental Inspection and Control (CICA). Before fixation in ethanol 99%, the fish were anesthetized using tricaine (MS222) for several minutes and then kept at 4°C for 15 min. Most of the individuals studied were voucher numbered and stored in the collection of the Museum Felipe Poey (MFP) of the University of Havana (Table 1).

**Table 1:** Sampling localities and number of *Lucifuga dentata* individuals analysed.

Cave number^a^	N^b^	Sampling locality(cave name, nearest village, municipality, province)	Cave abbreviation names	MFP Voucher number^c^
1	6	Judio, Guanahacabibes, Sandino, Pinar del Río	Jud	18.000048, 18.000195, 18.000312–18.000315
2	1	El Grillo, La Lima, Sandino, Pinar del Río	Gri	18.000316
3	2	La Raja, Majin, Sandino, Pinar del Río	Raj	18.000317 (2)
4	3	El Patrón, Jarreta, Sandino, Pinar del Río	Pat	18.000319 (3)
5	3	El Jagüey, Malpotón, Sandino, Pinar del Río	Jag	18.000318 (3)
6	4	Felipe, Cayuco, Sandino, Pinar del Río	Fel	18.000320 (4)
7	4	Pozo Azul, Cayuco, Sandino, Pinar del Río	Paz	18.000321–18.000324
8	4	Paredones, La Salud, Caimito, Havana	Par	18.000327–18.000330
9	3	La Lechuza, Las Cañas, Artemisa, Havana	Lec	18.000345 (3)
10	7	Baño2, Las Cañas, Artemisa, Havana	Ban	18.000196, 18.000341(2), 18.000343 (4)
11	8	Emilio, Las Cañas, Artemisa, Havana	Emi	18.000340 (8)
12	2	El Sitio, Las Cañas, Artemisa, Havana	Sit	No voucher number
13	5	Juanelo Piedra, -, Quivicán, Havana	JPa	18.000332–18.000336
14	6	Chicharrones, -, Bolondrón, Matanzas	Chi	18.000347–18.000352
15	**13**	Pichi, -,Bolondrón, Matanzas	Pch	18.000369 (13)
16	**4**	Los Chivos, -, Bolondrón, Matanzas	Vos	18.000370 (4)
17	1	La Yagruma, -, Agramante, Matanzas	Yag	No voucher number
18	4	Perico Sánchez, -, Jagüey Grande, Matanzas	PSa	18.000197, 18.000353–18.000355
19	3	El Pozo, -, Agramante, Matanzas	Pzo	18.000356–18.000358
20	4	La Carreta, -, Agramante, Matanzas	Car	18.000362 (2), 18.000363 (2)
21	3	La Ratonera, -, Agramonte, Matanzas	Rat	18.000364–18.000366

a: See Fig 1 for geographic positions.

b: Additional localities sampled in the study are shown in bold, i.e. localities were– 11 new samples from Pichi and 3 from Los Chivos caves were taken.

c: Number in parenthesis indicates the number of individuals identified by the same voucher number. MFP: Museum Felipe Poey

N: sample size

### DNA extraction, amplification and sequencing

Small pieces of fin or muscle tissue (2–3 mm^3^) were digested with proteinase K (100 μg/mL) in 200 μL lysis buffer (100 mM Tris-HCl, pH 8.0, 10 mM EDTA, 100 mM NaCl, 0.1% SDS, and 50 mM DTT) at 55°C with slow constant shaking, followed by a phenol: chloroform extraction using Phase Lock Gel^TM^ (Eppendorf) tubes. The polymerase chain reaction (PCR) was used to amplify partial sequences of two regions of the mtDNA: 810 bp of the *cyt*b gene (for 14 individuals) with the primers Glufish (5' CCAATGACTTGAAGAACCACCGTTG) [20] and CB3Luc (5' TGCGAAGAGGAAGTACCATTC) [10], and the control region (407 bp) (for the 90 individuals), using the primers: CRA (5’ AATTCTCACCCCTAGCTCCCAAAG) and CRE (5’ CCTGAAGTAGGAACCAGATG) [21]. Five to 100 ng of DNA were used as template for a 30 μL PCR reaction containing one unit of GoTaq DNA polymerase (Promega), 0.2 nM of each primer, 0.2 μM dNTPs, and 1.5 mM MgCl_2_. PCR products were purified using the QIAquick^®^ PCR purification kit (QIAGEN) and cycle-sequenced in both directions using the Big Dye terminator sequencing kit (Applied Biosystems). The fragments were resolved on an ABI 3100 automated sequencer (Applied Biosystems). The *cyt*b sequences were deposited in the EMBL database with accession numbers FR716716-FR716717 and FR716721 for *cyt*b sequences and accession numbers FR873577 to FR873609 for the CR sequences. The *cyt*b sequences of 76 individuals previously analyzed [10] (accession numbers: FR716685–FR716687, FR716689, FR716691–FR716693, FR716695–FR716706, FR716708–FR716721) were also included in the analysis.

### Sequence editing and analyses

Sequences were edited using Bioedit Sequence Alignment Editor v. 5.0.9 [22] and manually corrected. The *cyt*b and CR segments were concatenated and aligned using MEGA6 [23]. To test for neutrality, Tajimas’s *D* statistics [24] was calculated using the program DnaSP v. 5.10 [25]. Statistical signification was obtained by 10,000 coalescence simulations.

### Spatial structure of genetic variation

We first explored the spatial structure of genetic variation using a Spatial Analysis of Molecular Variance (SAMOVA) [26]. SAMOVA defines groups of samples that are geographically homogeneous and also maximally differentiated from each other. One hundred simulated annealing processes were used for each value of K (number of groups). The SAMOVA was run from K = 2 to the value of K that maximises the value of the *F*_*CT*_ statistic. The level of the haplotype diversity (*h*), nucleotide diversity (π), and genetic differentiation (*F*_*ST*_) were estimated for the inferred groups (populations) using the program ARLEQUIN v. 3.5 [27].

We constructed a haplotype network using the program TCS v 1.21 [28], to visualize haplotype relationships. The analysis was conducted using 95% parsimoniously plausible branch connections between haplotypes.

### Model based comparison of phylogeographic scenarios using ABC

**Fig 2:**
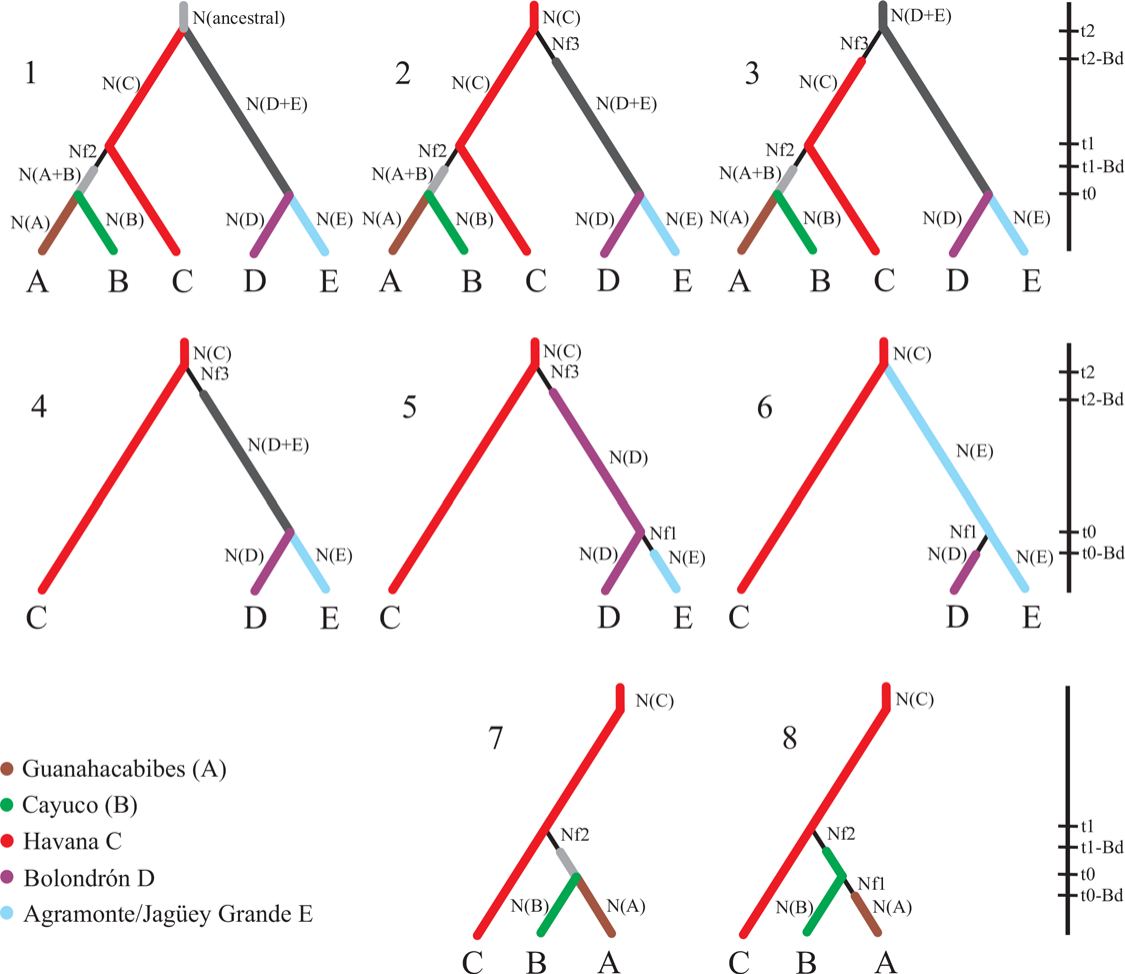
Competing scenarios set to test fragmentation and dispersal events in *L*. *dentata*. Dispersal events are followed by bottlenecks of short duration (thin lines). The main groups identified were: A (Guanahacabibes), B (Cayuco and surroundings), both in Pinar del Río; C (Havana); D (Bolondrón), E (Agramonte-Jagüey Grande) both in Matanzas. Bd indicates bottleneck event duration. Nf is the effective number of founders. N is the effective population size. Based on genetic diversity and demographic estimates the effective population sizes were set as follow: N(ancestral) was higher than the N of the derived populations and N(C) and N(E), in that order, were higher than the N of the other populations (A, B and D). Times when splits occurred were set to t2 > t1 > t0, based on the number of mutational steps in the network genealogy.

To evaluate the probability of different scenarios of dispersion and/or fragmentation accounting for the observed geographic pattern of genetic variation in *L*. *dentata*, we used the Approximate Bayesian Computation (ABC) method as implemented in the program DIYABC v. 1.0.4.46 [29]. The choice of scenarios was based on the identification of geographic genetic groups by SAMOVA and the architecture of the haplotype network. Information on the discontinuity of the karstic regions that could be useful in determining if fragmentation or dispersion had occurred, and the demographic reduction that most likely occurred just after colonisation (Fig 2), were included as priors in the various scenarios. The prior settings are shown in S1 Table.

In the first scenario an ancestral population, inhabiting the continuous southern karst of Matanzas and Havana regions (Fig 1) split, at a given time, into two geographical groups as a result of a fragmentation event, followed by a range expansion (*i*.*e*. long distance dispersal) to the west (*i*.*e*. from Havana to Pinar del Río). This is the inferred NCPA scenario from a previous analysis [10]. In the second scenario Havana was considered to be the ancestral population, from which two dispersals events occurred: an earlier one to the east and a second one to the west. This scenario was based on the topology of the haplotype network. The third scenario assumed that the source population was in Matanzas and successive dispersal events occurred to the west (a stepping-stone dispersal model from Matanzas to Havana and from Havana to Pinar del Río) (Fig 2). A direct Matanzas to Pinar del Río dispersal scenario was discarded because the topology of the haplotype network ruled out this possibility. A western (Pinar del Río) origin was discarded based on geological evidence (see Discussion). Dispersal and fragmentation events at smaller geographic scales were set for each scenario. In all cases it was assumed that when a dispersal event occurred, the new founder population passed through a bottleneck (Fig 2; S1 Table). At smaller scales (Cayuco–Guanahacabibes in Pinar del Río and Agramonte/Jagüey Grande–Bolondrón in Matanzas) (Fig 2) we tested both for dispersal and fragmentation using Havana as the ancestral population. The scenarios were set as described above.

The reference table for the scenarios was obtained after six million simulations of the data. This procedure was repeated three times to test for consistency of the results. The posterior probabilities of the competing scenarios were estimated using the logistic regression method [30] from 10% of the simulated data. The summary statistics (*i*.*e*. numbers of haplotypes, number of segregating sites, mean pairwise differences and variance of the pairwise differences) were chosen according to their accuracy and relevance for the parameters of interest for model estimation. We performed six million simulations in order to choose the set of summary statistics that best captured relevant aspects of the data [31, 32, 33]. *F*_*ST*_ statistics and mean pairwise genetic differences within and between groups were also calculated [34].

### Demographic inferences

To test for demographic changes (*i*.*e*. population expansion) a mismatch distribution analysis of pairwise nucleotide differences between haplotypes was performed using ARLEQUIN v. 3.5 [34]. The goodness of fit of the observed distribution with respect to the expected values was determined by the method of sum of square deviations (SSD) [35]. Additionally, we estimated the neutrality statistics *Fs* [36] and *R2* [37], which are sensitive to demographic changes. The statistics and their statistical significance, estimated by 10,000 coalescence simulations, were obtained using DnaSP v. 5.10 [25].

## Results

### Sequence variation

The total length of the concatenated *cyt*b and CR sequences is 1,217 bp. The alignment has 82 variable sites (40 within the *cyt*b sequence and 41 within the CR sequence) comprised of one indel, 65 transitions (TS), 11 tranversions (TV) and 5 sites with both TS and TV (TS/TV = 4.75). A total of 49 haplotypes were identified (see S2 Table for frequencies and geographic distribution). The Tajima´s *D* statistic showed no significant departure from zero (*D* = -0.841, p > 0.05), as expected under neutral sequence evolution.

### Identification of geographic groups

The SAMOVA results showed that *F*_*CT*_ statistic steadly increased as the number of groups of populations increased, from 0.514 for two groups, up to 0.745 for 12 groups (data not shown). Populations included individuals of single caves (seven instances), two caves (Baño + Emilio; Lechuza + Sitio; Perico Sanchez + Yagruma), three caves (Chicharrones + Chivo + Pichi) and a group of five caves (Felipe + Pozo Azul + Patrón + El Jagüey + La Raja) in each case, relatively close geographically (see Fig 1 and Table 1).

The network obtained with *cyt*b + CR sequences is shown in Fig 1. Compared with the network obtained previously with the *cyt*b sequences only [10], the number of mutation steps between regional groups of haplotypes increased, whereas the number of changes within the regions did not. For example, a minimum of 10 mutation steps distinguishes the Havana and Matanzas haplotypes groups compared to one mutation step in the network inferred using *cyt*b sequences (see Fig 1, and S2 in García-Machado, et al. [10]). As previously shown, most haplotypes have a restricted distribution. Eight haplotypes are shared by geographically close caves (distances between caves ranging from few meters to around 30 km) and two haplotypes (LdH23 and LdH24), genealogically related with Matanzas’s haplotypes, were found in Havana [10] (Fig 1, S2 Table) and are evidence for a relatively recent migration event.

As the number of available samples was low for several caves (n = 1 to 4, see Fig 1), subsequent analyses testing hypotheses regarding geographic distribution were conducted after grouping geographically close caves, based on network topology, geography and the groups of populations defined by SAMOVA. We identified five population groups *F*_*CT*_ = 0.656) that represent the major geographic cores: Guanahacabibes (extreme West) and Cayuco (West), in Pinar del Río; Havana (middle); Bolondrón (East) and Agramonte/Jagüey Grande (extreme East), in Matanzas (see Fig 1). Pinar del Río (except el Grillo cave) and Bolondrón localities represent two groups with a few high frequency haplotypes shared by several caves. The Guanahacabibes area (with a unique sample locality) was also recovered as a distinct group. Most of the localities identified as a single group have diagnostic haplotypes (*i*.*e*. in Agramonte/Jagüey Grande region), but because of the small sample sizes per locality (and the close relationships of haplotypes among neighbouring localities, see S2 Table), we cannot rule out the possibility of shared haplotypes between geographically related localities. As a general pattern, haplotypes within a geographic region were more closely related than between regions, and were distinguished by diagnostic nucleotide sites (S2 Table).

### Genetic diversity

The genetic diversity estimates for the five groups of populations are summarised in Table 2. Overall, the haplotype diversity (*h* = 0.979 ± 0.005) and nucleotide diversity (π = 0.01 ± 0.005) were high. The group from Havana has the highest haplotype diversity (*h* = 0.924) and nucleotide diversity (π = 0.0065; t-test = 3.6, p < 0.05 with respect to the second highest value). The other four populations also had relatively high *h* values, with the exception of Guanahacabibes (El Judio cave; *h* = 0,600, t-test = -5.08, p < 0.05), but had significant lower values of nucleotide diversity as shown above (Table 2). The pairwise population differentiation estimates (*F*_*ST*_) were also very high (*F*_*ST*_ ranging from 0.46 to 0.91) and highly significant for all population comparisons (S3 Table).

**Table 2:** Genetic diversity in *Lucifuga dentata*.

Population group	N	S	NH	*h*	π
Guanahacabibes	6	2	2	0.600 ± 0.129	0.0010 ± 0.0008
Cayuco	17	18	11	0.882 ± 0.072	0.0021 ± 0.0013
Havana	29	42	16	0.924 ± 0.030	0.0065 ± 0.0034
Bolondrón	23	12	11	0.909 ± 0.034	0.0023 ± 0.0014
Agramonte/Jagüey Grande	15	15	9	0.924 ± 0.044	0.0035 ± 0.0021
Overall	90	81	49	0.979 ± 0.005	0.0100 ± 0.0050

N: number of individuals; S: number of segregating sites; NH: number of haplotypes; *h*: haplotype diversity; π: nucleotide diversity; ±: standard deviation.

### Tests of phylogeographic scenarios

The results of the evaluation of scenarios 1, 2 and 3 (Fig 2) for the three main regions using the Approximate Bayesian Computation (ABC) method are show in Table 3. The computations supported, with the highest mean probability of 82% (range 79 to 83%) the East to West dispersion scenario (scenario 3). The alternative scenarios were either much less well supported, dispersion from Havana to East and West (scenario 2) had a mean probability of 18% (range 17 to 21%); or not supported at all, as for a past population split between Havana-Matanzas regions and dispersion from Havana to Pinar del Río (scenario 1). At the intra-regional scale, the test supported dispersal as the most likely process between the Agramonte/Jagüey Grande and Bolondrón regions but could not resolve the direction. However, a slightly higher probability was consistently assigned to Agramonte/Jagüey Grande (eastern) as the source population (48 to 53% versus 34 to 38% for the opposite direction). Finally, between Guanahacabibes and Cayuco, fragmentation and dispersion were equally likely (Table 3).

**Table 3:** Posterior probabilities for various scenarios of dispersion and/or fragmentation in *Lucifuga dentata*. See Fig 2 for scenario representations.

Tested scenarios	Range of posterior probabilities (mean) for 3 runs
*Major geographic regions*	
1 (fragmentation Havana—Matanzas)	0
2 (Havana origin and dispersion to both East and West)	0.17–0.21 (0.18)
3 (Matanzas origin and dispersion from East to West)	0.79–0.83 (0.82)
*Matanzas region*	
4 (fragmentation Agramonte/Jagüey Grande—Bolondrón)	0.13–0.15 (0.14)
5 (Bolondrón origin and dispersion to Agramonte/Jagüey Grande)	0.34–0.38 (0.36)
6 (Agramonte/Jagüey Grande origin and dispersion to Bolondrón)	0.48–0.53 (0.50)
*Pinar del Río region*	
7 (fragmentation in Pinar del Río)	0.44–0.54 (0.49)
8 (Cayuco origin and dispersion to Guanahacabibes)	0.46–0.56 (0.51)

### Demographic inferences

The mismatch distribution analysis showed a good fit between the expected and observed distributions of pairwise differences for the population of Agramonte / Jagüey Grande, Bolondrón and Cayuco, suggesting population expansion for this group (S1 Fig). However, although the other two populations (Guanahacabibes and Havana) had multimodal distributions, the SSD test did not reject a possible sudden expansion (Table 4).

**Table 4:** Demographic parameters in the five *Lucifuga dentata* haplogroups.

Population group	N	τ	θ_0_	SSD	*R2*	*Fs*
Guanahacabibes	6	2.42	0.00	0.233 NS	0.300 NS	1.938 NS
Cayuco	17	1.72	0.00	0.009 NS	0.083 *	-5.736 ***
La Havana	29	5.89	2.36	0.021 NS	0.085 NS	-2.072 NS
Bolondrón	23	3.12	0.10	0.003 NS	0.097 NS	-3.540 *
Agramonte/ Jagüey Grande	15	5.44	0.00	0.005 NS	0.129 NS	-1.572 NS

N: number of individuals, τ: time since the expansion in units of mutational time, θ_0_: pre-expansion values for the mutation parameter, SSD: sum of square deviations, *R2*: Ramos-Onsins and Rozas [37], *Fs*: Fu [36]. The probability of obtaining values lower or equal than the observed value after 10,000 coalescence simulations are represented by NS: Not significant

*: p < 0.05

***: p < 0.001.

The *R*_2_ and *F*_*S*_ statistics were negative and statistically significant for Cayuco and Bolondrón (Table 4), supporting population expansion in these cases. For the other populations (Agramonte / Jagüey Grande, Guanahacabibes, and Havana), no significant departures from equilibrium values were observed (Table 4).

## Discussion

For most troglobitic species, dependence on very narrow environmental conditions has lead to a high degree of divergence between populations inhabiting geograpically proximate caves [9, 11, 13, 14, 15]. Nevertheless, under favourable circumstances, some organisms can circumvent geographic barriers, disperse and expand their distribution [7, 9, 16, 17, 18].

In the present work, using additional sequences and samples, we provide further details on the phylogeography of the blind cavefish *L*. *dentata*. The use of probabilistic methods of inference allowed us to deduce the most likely process that has shaped the present population distribution.

### Dispersion versus fragmentation

The present results indicate that *L*. *dentata* has a more complex geographic population structure than previously recognized from a *cyt*b analysis alone. We have identified five genetic and geographically concordant groups, two more than previously inferred [10]: two in the Pinar del Río province (Cayuco and Guanahacabibes), one in the Havana province and two in the Matanzas province (Agramonte/Jagüey Grande and Bolondrón). This result was mainly due to the inclusion of CR sequences which provided additional polymorphic sites, improving regional haplotype group delimitations. No haplotypes were shared amongst the main groups (S2 Table) and most haplotypes (41 of 49) were diagnostic of a given cave (S2 Table). Unique haplotypes in several caves also indicates high population isolation within each group. Similar patterns, which suggest very restricted or no gene flow between caves, have been identified in other stygobitic organisms, including salamanders [38], beetles [39], crickets [40], amphipods [13, 14, 41], isopods [42], shrimps [15] as well as other fishes such as *Schistura oedipus* [43] and *Astyanax mexicanus* [5, 6, 44].

The approach (ABC) used to infer the processes that shaped the current distribution of the genetic variability in *L*. *dentata* suggested sequential dispersal and settlement from the Matanzas region to the west. Published information on subterranean hydrological networks in Cuba is scarce and this makes it difficult to use additional evidence to support the likelihood of one or another inferred processes. However, other indirect evidence supports a stepping stone model of dispersion as the most likely process resulting in the present distribution of *L*. *dentata* as well as other members of this genus. Haplotype distributions suggest that continuity of karst connections varies from one region to another. For instance, individuals from the five sampled caves in the Agramonte / Jagüey Grande region all have diagnostic haplotypes, indicating, at least for relatively recent times, there is very low migration rates between caves separated by only a few kilometres (3 to 7 km). In contrast, the Bolondrón, Havana and Pinar del Río (excluding El Judio) regions have haplotypes that are shared between intraregional caves separated in several cases by larger karst gaps, suggesting that migrations occur more frequently between these caves. At the scale of the entire *L*. *dentata* distribution, the five major regions do not share haplotypes, indicating no exchange of individuals between these populations in recent times. However, the occurrence of haplotypes in Havana genealogically derived from the Matanzas region, and relatively divergent from their most closely related haplotype, is the signature of a more ancient dispersal event (Fig 1). These contrasting patterns support the view that dispersal between underground aquifers does not only depend on the ability of the organism to disperse, but also on the complexity of the underground water network [9, 15, 18, 39, 45] as well as barriers that under certain hydrological conditions (*e*.*g*. raise of underground water levels) can be overcome, facilitating transient migration between distant regions [6, 7, 15, 44].

The origin of *L*. *dentata* in southern Matanzas karst and its inferred westward dispersal is further supported by the identification of sister species in the north of this region [10]. Phylogeographic inferences indicate a second and relatively more recent dispersal event from Havana to the Pinar del Río province. The haplogroup from Cayuco and nearby areas show signatures of demographic expansion after a population bottleneck or recent founder event, such as a star like haplotype network (Fig 1) [46] and significant values of *Fs* and *R2* statistics (Table 4; S1 Fig).

At a smaller geographic scale, two other recent simultaneous events (*i*.*e*. same number of mutations between groups) were inferred in Matanzas between Bolondrón and Agramonte/Jagüey Grande, and in Pinar del Río between Cayuco and Guanahacabibes. In both cases dispersal events appear to have shaped current distributions of genetic variation. Although neither fragmentation nor dispersion between Cayuco and Guanahacabibes were estimated as being more probable in the ABC analyses (see Table 2) paleogeographic evidence suggests that the Guanahacabibes peninsula is one of the last land masses that emerged from the sea [47, 48] and it is possible that colonization of this area was from a population in neighbouring regions above the sea (*i*.*e*. Cayuco and surrounding areas).

### Range expansion

Compared to other Cuban *Lucifuga* species, *L dentata* is one of the most demographically and geographically successful. That is, this species has relatively high levels of genetic diversity (overall mean *h* = 0.979 ± 0.005, and π = 0.01 ± 0.005) as well as a large geographic distribution. Direct field observations suggest that this species’ census size is larger than that of others *Lucifuga* species inhabiting the same caves. The relative numbers of *L*. *dentata* and *L*. *subterranea* in Havana caves and the relative numbers of *L*. *dentata* and *Lucifuga* sp. 3 in Pozo Azul (Cayuco region) are about ten to one or more. The geographic distribution of genetic variation in *L*. *dentata* appears most compatible with a historical scenario of a one way stepping stone dispersal from East to West followed by strong isolation, in sharp contrast with the very narrow distribution of the other Cuban *Lucifuga* species. Overall, this study highlights the importance of genetic data as indirect evidence for underground connectivity patterns, in particular when direct data are unavailable.

The ecological characteristics of a freshwater fish species can be used to predict its dispersal capabilities [49]. Therefore, it would be interesting to study the biological properties that could increase the dispersion capabilities and range expansion of this particular species. One approach would be to compare the population dynamics (including demographic changes, predation, and births rates) in caves inhabited by *L*. *dentata* and another *Lucifuga* species.

Finally, the high genetic diversity and relatively large distribution of *L*. *dentata* had lead to this species being considered as not threatened in the Red List of Cuban Vertebrates [50]. Nonetheless, strong isolation of most cave populations does make this species vulnerable. The intensive use of subterranean water and the introduction of exotic species (e.g. *Clarias gariepinus*) in some aquifers are the main factors of concern.

## Supporting Information

S1 Fig Mismatch distribution of pairwise haplotype differences for the *cyt*b+NCR from *Lucifuga dentata*. Diamonds connected by dash represent observed distributions; continuous and broken lines indicate expected distributions and confidence intervals respectively.
(DOCX)

S1 Table Prior parameter distribution used for the ABC model analysis of the *Lucifuga dentata* data. See Fig 2 for graphical representation of the tested scenarios.
(DOC)

S2 Table A. Number and geographic distribution of the *cytb*+NCR haplotypes of *Lucifuga dentata*. B. Variable sites characterizing the 49 *cytb*+NCR haplotypes of *Lucifuga dentata*.
(DOC)

S3 Table Mean pairwise genetic differentiation (*F*_*ST*_) estimates between the five geographic groups of populations. (DOCX)
