# Supplementary material for: Go West: A One Way Stepping-Stone Dispersion Model for the Cavefish Lucifuga dentata in Western Cuba
Source: PLoS One. 2016 Apr 15;11(4):e0153545. doi: 10.1371/journal.pone.0153545 (PMC4833296; doi:10.1371/journal.pone.0153545)

**S1 Fig**

Mismatch distribution of pairwise haplotype differences for the *cyt*b+NCR from *Lucifuga dentata*. Diamonds connected by dash represent observed distributions; continuous and broken lines indicate expected distributions and confidence intervals respectively.


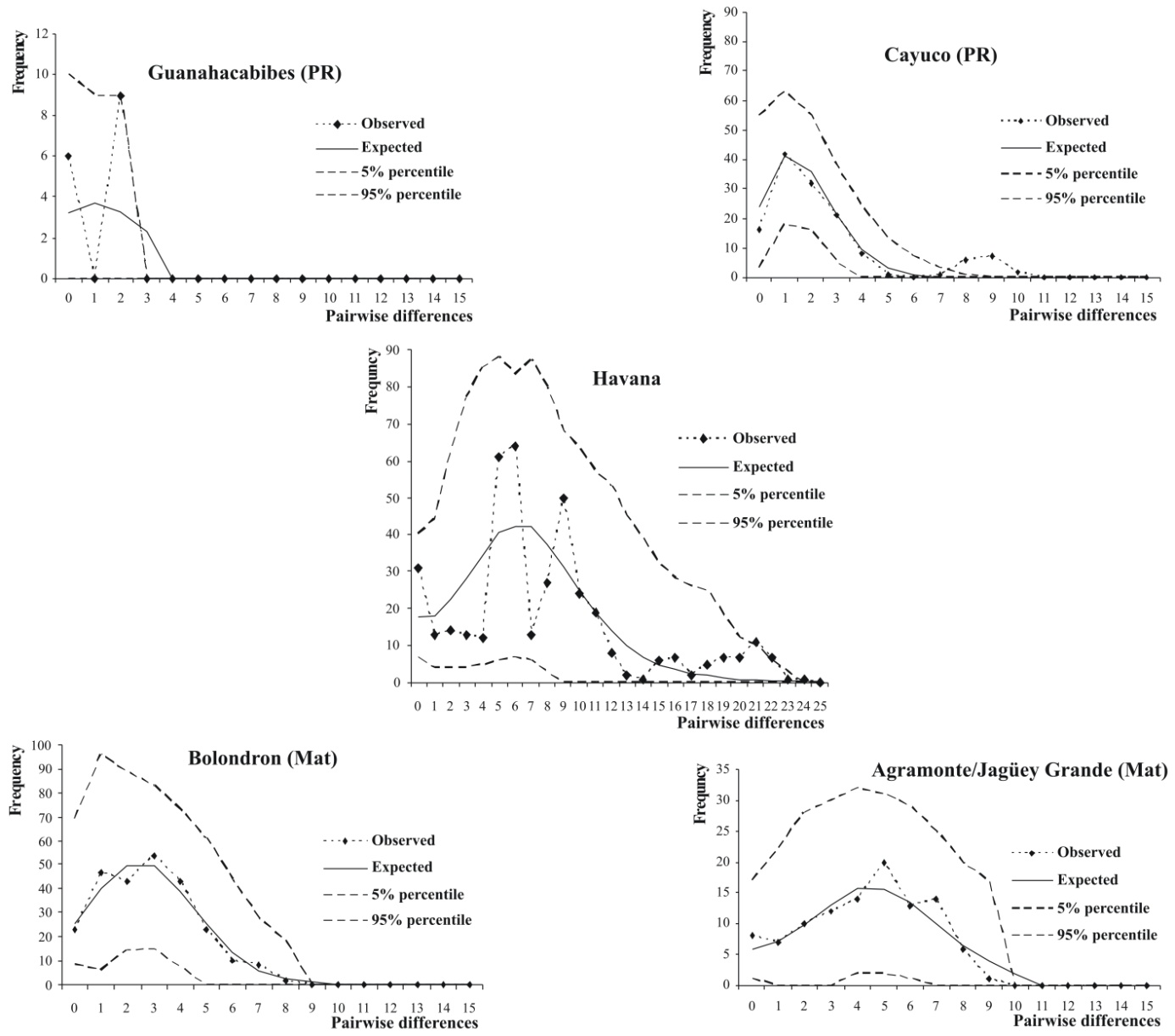

Supplement: S1 Fig — Diamonds connected by dash represent observed distributions; continuous and broken lines indicate expected distributions and confidence intervals respectively. (DOCX) [file pone.0153545.s001.docx]
