# Supplementary material for: Go West: A One Way Stepping-Stone Dispersion Model for the Cavefish Lucifuga dentata in Western Cuba
Source: PLoS One. 2016 Apr 15;11(4):e0153545. doi: 10.1371/journal.pone.0153545 (PMC4833296; doi:10.1371/journal.pone.0153545)
Supplement: S1 Table — See Fig 2 for graphical representation of the tested scenarios. (DOC) [file pone.0153545.s002.doc]

**S1 Table**

Prior parameter distribution used for the ABC model analysis of the *Lucifuga dentata* data. See Figure 2 for graphical representation of the tested scenarios.

| Demographic Parameters | Description | Parameter prior intervals* |
| --- | --- | --- |
| N(A) | Guanahacabibes group effective population size | 10-10000 |
| N(B) | Pinar del Río group effective population size | 10-10000 |
| N(C) | La Havana group effective population size | 10-10000 |
| N(D) | Bolondrón group effective population size | 10-10000 |
| N(E) | Agramonte group effective population size | 10-10000 |
| N(Ancestral) | Ancestral effective population size | 10-10000 |
| t | Divergence time (in generations) | 1-10000 |
| Nf | Effective number of founders | 1-200 |
| Bd | Bottleneck duration (in generations) | 1-3000 |
| Mutation |  |  |
| *μ_snp_* | Mutation rate for mtDNA | 1x10^-8^-1x10^-7^ |

*Uniform distribution was assumed for parameters and fixed parameter minimum and maximum relatively wide.
