# Supplementary material for: Go West: A One Way Stepping-Stone Dispersion Model for the Cavefish Lucifuga dentata in Western Cuba
Source: PLoS One. 2016 Apr 15;11(4):e0153545. doi: 10.1371/journal.pone.0153545 (PMC4833296; doi:10.1371/journal.pone.0153545)
Supplement: S2 Table — A. Number and geographic distribution of the cytb+NCR haplotypes of Lucifuga dentata. B. Variable sites characterizing the 49 cytb+NCR haplotypes of Lucifuga dentata. (DOC) [file pone.0153545.s003.doc]

**S2 Table**

A. Number and geographic distribution of the *cytb*+NCR haplotypes of *Lucifuga dentata*.

| Region | Pinar del Río | | | | | | | La Havana | | | | | | Matanzas | | | | | | | |
| --- | --- | --- | --- | --- | --- | --- | --- | --- | --- | --- | --- | --- | --- | --- | --- | --- | --- | --- | --- | --- | --- |
| Cave  Haplot. | Jud | Gri | Raj | Pat | Jag | PAz | Fel | Emi | Ban | Lec | Sit | Par | JPa | Chi | Vos | Pch | PSa | Pzo | Yag | Car | Rat |
| LdH1 | 3 |  |  |  |  |  |  |  |  |  |  |  |  |  |  |  |  |  |  |  |  |
| LdH2 | 3 |  |  |  |  |  |  |  |  |  |  |  |  |  |  |  |  |  |  |  |  |
| LdH3 |  |  |  | 1 |  |  |  |  |  |  |  |  |  |  |  |  |  |  |  |  |  |
| LdH4 |  |  |  |  | 1 |  |  |  |  |  |  |  |  |  |  |  |  |  |  |  |  |
| LdH5 |  |  |  |  | 1 |  |  |  |  |  |  |  |  |  |  |  |  |  |  |  |  |
| LdH6 |  |  |  |  |  |  | 1 |  |  |  |  |  |  |  |  |  |  |  |  |  |  |
| LdH7 |  |  | 1 |  |  |  |  |  |  |  |  |  |  |  |  |  |  |  |  |  |  |
| LdH8 |  |  |  | 1 | 1 | 3 | 1 |  |  |  |  |  |  |  |  |  |  |  |  |  |  |
| LdH8a |  |  |  |  |  |  | 2 |  |  |  |  |  |  |  |  |  |  |  |  |  |  |
| LdH8b |  |  |  | 1 |  |  |  |  |  |  |  |  |  |  |  |  |  |  |  |  |  |
| LdH9 |  |  | 1 |  |  |  |  |  |  |  |  |  |  |  |  |  |  |  |  |  |  |
| LdH10 |  | 1 |  |  |  |  |  |  |  |  |  |  |  |  |  |  |  |  |  |  |  |
| LdH11 |  |  |  |  |  | 1 |  |  |  |  |  |  |  |  |  |  |  |  |  |  |  |
| LdH12 |  |  |  |  |  |  |  |  |  |  |  |  | 1 |  |  |  |  |  |  |  |  |
| LdH13 |  |  |  |  |  |  |  |  |  |  |  |  | 1 |  |  |  |  |  |  |  |  |
| LdH14 |  |  |  |  |  |  |  |  |  |  |  | 1 |  |  |  |  |  |  |  |  |  |
| LdH15 |  |  |  |  |  |  |  |  |  |  |  | 3 | 2 |  |  |  |  |  |  |  |  |
| LdH16 |  |  |  |  |  |  |  | 4 | 1 | 1 |  |  |  |  |  |  |  |  |  |  |  |
| LdH17 |  |  |  |  |  |  |  |  |  | 1 |  |  |  |  |  |  |  |  |  |  |  |
| LdH18 |  |  |  |  |  |  |  | 1 | 2 |  |  |  |  |  |  |  |  |  |  |  |  |
| LdH18a |  |  |  |  |  |  |  | 1 |  |  |  |  |  |  |  |  |  |  |  |  |  |
| LdH18b |  |  |  |  |  |  |  |  | 1 |  |  |  |  |  |  |  |  |  |  |  |  |
| LdH19 |  |  |  |  |  |  |  |  | 1 |  |  |  |  |  |  |  |  |  |  |  |  |
| LdH20 |  |  |  |  |  |  |  | 1 |  |  |  |  |  |  |  |  |  |  |  |  |  |
| LdH21 |  |  |  |  |  |  |  |  | 1 |  | 1 |  | 1 |  |  |  |  |  |  |  |  |
| LdH21a |  |  |  |  |  |  |  | 1 |  |  |  |  |  |  |  |  |  |  |  |  |  |
| LdH22 |  |  |  |  |  |  |  |  | 1 |  |  |  |  |  |  |  |  |  |  |  |  |
| LdH23 |  |  |  |  |  |  |  |  |  | 1 |  |  |  |  |  |  |  |  |  |  |  |
| LdH24 |  |  |  |  |  |  |  |  |  |  | 1 |  |  |  |  |  |  |  |  |  |  |
| LdH25 |  |  |  |  |  |  |  |  |  |  |  |  |  |  |  |  |  |  |  |  | 3 |
| LdH26 |  |  |  |  |  |  |  |  |  |  |  |  |  |  |  |  | 3 |  |  |  |  |
| LdH26a |  |  |  |  |  |  |  |  |  |  |  |  |  |  |  |  |  |  |  | 1 |  |
| LdH27 |  |  |  |  |  |  |  |  |  |  |  |  |  |  |  |  |  |  |  | 2 |  |
| LdH27a |  |  |  |  |  |  |  |  |  |  |  |  |  |  |  |  |  |  |  | 1 |  |
| LdH28 |  |  |  |  |  |  |  |  |  |  |  |  |  |  |  |  | 1 |  |  |  |  |
| LdH29 |  |  |  |  |  |  |  |  |  |  |  |  |  |  |  |  |  | 2 |  |  |  |
| LdH30 |  |  |  |  |  |  |  |  |  |  |  |  |  |  |  |  |  | 1 |  |  |  |
| LdH32 |  |  |  |  |  |  |  |  |  |  |  |  |  |  |  | 3 |  |  |  |  |  |
| LdH32a |  |  |  |  |  |  |  |  |  |  |  |  |  |  |  |  |  |  | 1 |  |  |
| LdH33 |  |  |  |  |  |  |  |  |  |  |  |  |  | 2 | 1 | 2 |  |  |  |  |  |
| LdH33a |  |  |  |  |  |  |  |  |  |  |  |  |  | 1 |  | 3 |  |  |  |  |  |
| LdH33b |  |  |  |  |  |  |  |  |  |  |  |  |  |  |  | 3 |  |  |  |  |  |
| LdH33c |  |  |  |  |  |  |  |  |  |  |  |  |  | 1 |  |  |  |  |  |  |  |
| LdH33d |  |  |  |  |  |  |  |  |  |  |  |  |  | 1 |  |  |  |  |  |  |  |
| LdH33e |  |  |  |  |  |  |  |  |  |  |  |  |  |  | 1 |  |  |  |  |  |  |
| LdH34 |  |  |  |  |  |  |  |  |  |  |  |  |  |  | 1 |  |  |  |  |  |  |
| LdH35 |  |  |  |  |  |  |  |  |  |  |  |  |  |  |  | 1 |  |  |  |  |  |
| LdH36 |  |  |  |  |  |  |  |  |  |  |  |  |  | 1 |  |  |  |  |  |  |  |
| LdH37 |  |  |  |  |  |  |  |  |  |  |  |  |  |  | 1 | 1 |  |  |  |  |  |

Cave name abbreviations as in Table 1.

Haplotypes with same number follow by a letter refer to haplotypes derived from *cytb* haplotypes and defined in García-Machado *et al*. (2011).

B. Variable sites characterizing the 49 *cytb*+NCR haplotypes of *Lucifuga dentata*.

| Geographic Location | | | Haplotypes | Nucleotide Position | | | | | | | | | | | | | | | | | | | | | | | | | | | | | | | | | | | | | | | | | N |
| --- | --- | --- | --- | --- | --- | --- | --- | --- | --- | --- | --- | --- | --- | --- | --- | --- | --- | --- | --- | --- | --- | --- | --- | --- | --- | --- | --- | --- | --- | --- | --- | --- | --- | --- | --- | --- | --- | --- | --- | --- | --- | --- | --- | --- | --- |
|  | | |  | 0 | 0 | 0 | 1 | 1 | 1 | 1 | 2 | 2 | 3 | 3 | 3 | 3 | 3 | 3 | 3 | 3 | 4 | 4 | **4** | 4 | 4 | 5 | 5 | 5 | 6 | **6** | 6 | 6 | 6 | 6 | 6 | 6 | 6 | 7 | 7 | 7 | 7 | 7 | 7 |  |  |
|  | | |  | 0 | 0 | 1 | 0 | 7 | 9 | 9 | 6 | 7 | 1 | 2 | 5 | 6 | 6 | 6 | 6 | 9 | 3 | 4 | **7** | 8 | 9 | 6 | 6 | 7 | 0 | **2** | 3 | 4 | 5 | 7 | 8 | 9 | 9 | 2 | 3 | 3 | 4 | 4 | 8 |  |  |
|  | | |  | 6 | 7 | 9 | 8 | 7 | 7 | 9 | 4 | 6 | 8 | 7 | 4 | 0 | 3 | 6 | 7 | 0 | 4 | 7 | **3** | 2 | 8 | 4 | 7 | 4 | 6 | **5** | 6 | 8 | 6 | 4 | 7 | 5 | 7 | 5 | 5 | 9 | 6 | 8 | 4 |  |  |
|  | | |  | C | A | A | C | T | T | A | C | C | G | C | T | A | C | A | G | T | T | C | A | T | G | T | C | T | A | A | C | T | C | C | C | T | G | T | T | C | T | C | C |  |  |
| P | Guanaha | | LdH1 | . | . | . | . | . | . | . | . | . | . | . | . | . | . | . | . | . | . | . | . | . | . | . | . | . | . | **.** | . | . | . | . | . | . | . | . | . | . | . | . | . |  | 3 |
| i | cabibes | | LdH2 | . | . | . | . | . | . | . | . | . | . | . | C | . | . | . | . | . | . | . | . | . | . | . | . | . | . | **.** | . | . | . | . | . | . | . | . | . | . | . | . | . |  | 3 |
| n |  | | LdH3 | . | . | . | . | . | . | . | . | . | . | . | C | . | . | . | . | . | . | . | . | . | . | . | . | . | . | **.** | . | . | . | . | . | . | . | . | . | . | . | T | . |  | 1 |
| a |  | | LdH4 | . | . | . | . | . | C | . | . | . | . | . | C | . | . | . | . | . | . | . | . | . | . | C | . | . | . | **.** | . | . | . | . | . | . | . | . | . | . | . | T | . |  | 1 |
| r |  | | LdH5 | . | . | . | T | . | . | . | . | . | . | . | C | . | . | . | . | . | . | . | . | . | . | C | . | . | . | **.** | . | . | . | . | . | . | . | . | . | . | . | T | . |  | 1 |
|  | C | | LdH6 | . | . | . | . | . | . | . | . | . | . | . | C | . | . | . | . | . | . | . | . | . | . | C | . | . | . | **.** | . | . | . | . | T | . | . | . | . | . | . | T | . |  | 1 |
| d | a | | LdH7 | . | C | . | . | . | . | . | . | . | . | . | C | . | . | . | . | . | . | . | . | . | . | C | . | . | . | **.** | . | . | . | . | . | . | . | . | . | . | . | T | . |  | 1 |
| e | y | | LdH8 | . | . | . | . | . | . | . | . | . | . | . | C | . | . | . | . | . | . | . | . | . | . | C | . | . | . | **.** | . | . | . | . | . | . | . | . | . | . | . | T | . |  | 6 |
| l | u | | LdH8a | . | . | . | . | . | . | . | . | . | . | . | C | . | . | . | . | . | . | . | . | . | . | C | . | . | . | **.** | . | . | . | . | . | . | . | . | . | . | . | T | . |  | 2 |
|  | c | | LdH8b | . | . | . | . | . | . | . | . | . | . | . | C | . | . | . | . | . | . | . | . | . | . | C | . | . | . | **.** | . | . | . | . | . | . | . | . | . | . | . | T | . |  | 1 |
| R | o | | LdH9 | . | . | . | . | . | . | . | . | . | . | T | C | . | . | . | . | . | . | . | . | . | . | C | . | . | . | **.** | . | . | . | . | . | . | . | . | . | . | . | T | . |  | 1 |
| í |  | | LdH10 | . | . | . | . | . | . | . | . | . | . | T | C | . | . | . | . | . | . | . | . | . | . | C | . | . | . | **.** | . | . | . | . | . | . | . | . | . | . | . | . | . |  | 1 |
| o |  | | LdH11 | . | . | . | . | . | . | . | . | . | . | . | C | . | A | . | . | . | . | . | . | . | . | C | . | . | . | **.** | T | . | . | . | . | . | . | . | . | . | . | . | . |  | 1 |
|  | | | LdH12 | . | . | . | . | . | . | . | . | . | A | . | C | . | . | . | A | . | . | . | . | . | A | C | . | . | . | G | . | . | . | . | . | . | . | . | . | . | C | . | . |  | 1 |
|  | | | LdH13 | . | . | . | . | . | . | . | . | . | . | . | C | G | . | . | A | . | . | . | . | . | . | C | . | . | . | G | . | . | . | . | . | . | . | . | . | . | . | . | . |  | 1 |
|  | | | LdH14 | . | . | . | . | . | . | . | . | . | . | . | C | . | . | . | . | . | . | . | . | . | . | C | . | . | . | G |  | G | . | . | . | . | . | . | C | . | . | . | G |  | 1 |
|  | | | LdH15 | . | . | . | . | . | . | . | . | . | . | . | C | . | . | . | . | . | . | . | . | . | . | C | . | . | . | G | . | . | . | . | . | . | . | . | C | . | . | . | . |  | 5 |
|  | | | LdH16 | . | . | . | . | . | . | . | . | . | . | . | C | . | . | . | . | . | . | . | . | . | . | C | . | . | . | G | . | . | . | . | . | . | . | . | . | . | . | . | . |  | 6 |
| H | | | LdH17 | . | . | . | . | . | . | . | . | A | . | . | C | . | . | . | . | . | . | . | . | . | . | C | . | . | . | G | . | . | . | . | . | . | . | . | . | . | . | . | . |  | 1 |
| a | | | LdH18 | . | . | . | . | . | . | . | . | A | . | . | C | . | . | . | . | C | . | . | . | . | . | C | . | . | . | G | . | . | . | . | . | . | . | . | . | . | . | . | . |  | 3 |
| v | | | LdH18a | . | . | . | . | . | . | . | . | A | . | . | C | . | . | . | . | C | . | . | . | . | . | C | . | . | . | G | . | . | . | . | . | . | . | . | . | . | . | . | . |  | 1 |
| a | | | LdH18b | . | . | . | . | . | . | . | . | A | . | . | C | . | . | . | . | C | . | . | . | . | . | C | . | . | . | G | . | . | . | . | . | . | . | . | . | . | . | . | . |  | 1 |
| n | | | LdH19 | . | . | . | . | . | . | . | . | A | . | . | C | . | . | . | . | C | . | . | . | . | . | C | . | . | . | G | . | . | T | . | . | . | . | . | . | . | . | . | . |  | 1 |
| a | | | LdH20 | . | . | . | . | . | . | . | . | A | . | . | C | . | . | . | . | C | G | T | . | A | . | C | . | . | . | G | . | . | . | . | . | . | . | . | . | . | . | . | . |  | 1 |
|  | | | LdH21 | . | . | . | . | C | . | . | . | . | . | . | C | . | . | . | . | C | . | . | . | . | . | C | . | . | . | G | . | . | . | . | . | . | A | . | . | . | . | . | . |  | 3 |
|  | | | LdH21a | . | . | . | . | C | . | . | . | . | . | . | C | . | . | . | . | C | . | . | . | . | . | C | . | . | . | G | . | . | . | . | . | . | A | . | . | . | . | . | . |  | 1 |
|  | | | LdH22 | . | . | . | . | C | . | . | . | . | . | . | C | . | . | . | . | . | . | . | . | . | . | C | . | . | . | G | . | . | . | . | T | . | A | . | . | . | . | . | . |  | 1 |
|  | | | LdH23 | . | . | . | . | . | . | . | . | . | . | . | C | . | . | G | . | . | . | . | G | . | A | C | . | . | . | G | . | . | . | T | . | . | . | . | . | . | . | . | . |  | 1 |
|  | | | LdH24 | . | . | . | . | . | . | . | . | . | . | . | C | . | . | G | . | . | . | . | G | . | A | C | . | . | . | G | . | . | . | . | T | . | . | . | . | T | . | . | . |  | 1 |
|  | | A | LdH25 | . | . | . | . | . | . | . | . | . | . | . | C | . | . | . | . | . | . | . | **G** | . | . | C | . | . | . | G | . | . | . | . | . | . | . | . | . | . | . | . | . |  | 3 |
|  | | g | LdH26 | . | . | G | . | . | . | . | . | . | . | . | C | . | . | . | . | . | . | . | **G** | . | A | C | . | . | . | G | . | . | . | . | . | . | . | . | . | . | . | . | . |  | 3 |
|  | | r | LdH26a | . | . | G | . | . | . | . | . | . | . | . | C | . | . | . | . | . | . | . | **G** | . | A | C | . | . | . | G | . | . | . | . | . | . | . | . | . | . | . | . | . |  | 1 |
| M | | a | LdH27 | . | . | G | . | . | . | . | . | . | . | . | C | . | . | . | . | . | . | . | **G** | . | A | C | . | . | G | G | . | . | . | . | . | . | . | . | . | . | . | . | . |  | 2 |
| a | | m | LdH27a | . | . | G | . | . | . | . | . | . | . | . | C | . | . | . | . | . | . | . | **G** | . | A | C | . | . | G | G | . | . | . | . | . | . | . | . | . | . | . | . | . |  | 1 |
| t | | o | LdH28 | . | . | . | . | . | . | . | . | . | . | . | C | . | . | . | . | . | . | . | **G** | . | A | C | . | . | . | G | . | C | . | . | . | . | . | C | . | . | . | . | . |  | 1 |
| a | | n | LdH29 | . | . | . | . | . | . | G | . | . | . | . | C | . | . | . | . | . | . | . | **G** | . | A | C | . | . | . | G | . | . | . | . | . | . | . | . | . | . | . | . | . |  | 2 |
| n | | t | LdH30 | . | . | . | . | . | . | . | T | . | . | . | C | . | . | . | . | . | . | . | **G** | . | A | C | . | . | . | G | . | . | . | . | . | . | . | . | . | . | . | . | . |  | 1 |
| z | | e | LdH32a | . | . | . | . | . | . | . | . | . | . | . | C | . | . | . | . | . | . | . | **G** | . | A | C | . | . | . | G | . | . | . | . | . | . | . | . | . | . | . | . | . |  | 1 |
| a | |  | LdH32 | . | . | . | . | . | . | . | . | . | . | . | C | . | . | . | . | . | . | . | **G** | . | A | C | . | . | . | G | . | . | . | . | . | . | . | . | . | . | . | . | . |  | 3 |
| s | | B | LdH33 | . | . | . | . | . | . | . | . | . | . | . | C | . | . | . | . | . | . | . | **G** | . | A | C | T | . | . | G | . | . | . | . | . | . | . | . | . | . | . | . | . |  | 5 |
|  | | o | LdH33a | . | . | . | . | . | . | . | . | . | . | . | C | . | . | . | . | . | . | . | **G** | . | A | C | T | . | . | G | . | . | . | . | . | . | . | . | . | . | . | . | . |  | 4 |
|  | | l | LdH33b | . | . | . | . | . | . | . | . | . | . | . | C | . | . | . | . | . | . | . | **G** | . | A | C | T | . | . | G | . | . | . | . | . | . | . | . | . | . | . | . | . |  | 3 |
|  | | o | LdH33c | . | . | . | . | . | . | . | . | . | . | . | C | . | . | . | . | . | . | . | **G** | . | A | C | T | . | . | G | . | . | . | . | . | . | . | . | . | . | . | . | . |  | 1 |
|  | | n | LdH33d | . | . | . | . | . | . | . | . | . | . | . | C | . | . | . | . | . | . | . | **G** | . | A | C | T | . | . | G | . | . | . | . | . | . | . | . | . | . | . | . | . |  | 1 |
|  | | d | LdH33e | . | . | . | . | . | . | . | . | . | . | . | C | . | . | . | . | . | . | . | **G** | . | A | C | T | . | . | G | . | . | . | . | . | . | . | . | . | . | . | . | . |  | 1 |
|  | | r | LdH34 | . | G | . | . | . | . | . | . | . | . | . | C | . | . | . | . | . | . | . | **G** | . | A | C | T | . | . | G | . | . | . | . | . | . | . | . | . | . | . | . | . |  | 1 |
|  | | ó | LdH35 | . | . | . | . | . | . | . | . | . | . | . | C | . | . | . | . | . | . | . | **G** | . | A | C | T | . | . | G | . | . | . | . | . | C | . | . | . | . | . | . | . |  | 1 |
|  | | n | LdH36 | G | . | . | . | . | . | . | . | . | . | . | C | . | . | . | . | . | . | . | **G** | . | A | C | . | . | . | G | . | . | . | . | . | . | . | . | . | . | . | . | . |  | 1 |
|  | |  | LdH37 | . | . | . | . | . | . | . | . | . | . | . | C | . | . | . | . | . | . | . | **G** | . | A | C | . | C | . | G | . | . | . | . | . | . | . | . | . | . | . | . | . |  | 2 |
| S/V | | |  | V | ? | S | S | S | S | S | S | V | S | S | S | S | V | S | S | S | V | S | **S** | V | S | S | S | S | S | **S** | S | ? | S | S | S | S | S | S | S | S | S | S | V |  |  |
| Codon position | | |  | 3 | 1 | 1 | 3 | 3 | 2 | 1 | 3 | 3 | 3 | 3 | 3 | 3 | 3 | 3 | 1 | 3 | 2 | 3 | **2** | 2 | 3 | 3 | 3 | 1 | 3 | **1** | 3 | 3 | 2 | 2 | 3 | 2 | 1 | 2 | 3 | 1 | 2 | 1 | 1 |  |  |

B cont.

| Geographic Location | | | Haplotype | Nucleotide Position | | | | | | | | | | | | | | | | | | | | | | | | | | | | | | | | | | | | | | | | | N |
| --- | --- | --- | --- | --- | --- | --- | --- | --- | --- | --- | --- | --- | --- | --- | --- | --- | --- | --- | --- | --- | --- | --- | --- | --- | --- | --- | --- | --- | --- | --- | --- | --- | --- | --- | --- | --- | --- | --- | --- | --- | --- | --- | --- | --- | --- |
|  |  | |  |  |  |  |  |  |  |  |  |  |  |  |  |  |  |  |  |  | **1** | 1 | **1** | 1 | 1 | 1 | 1 | 1 | **1** | 1 | 1 | 1 | **1** | 1 | 1 | 1 | 1 | 1 | 1 | 1 | 1 | 1 | **1** | **1** |  |
|  |  | |  | 8 | **8** | **8** | 8 | 8 | **9** | 9 | **9** | 9 | 9 | 9 | 9 | 9 | **9** | 9 | 9 | 9 | **0** | 0 | **0** | 0 | 0 | 0 | 0 | 0 | **0** | 0 | 0 | 0 | **0** | 0 | 0 | 0 | 1 | 1 | 1 | 1 | 1 | 1 | **1** | **1** |  |
|  |  | |  | 2 | **2** | **3** | 3 | 8 | **0** | 1 | **1** | 3 | 3 | 3 | 3 | 6 | **8** | 8 | 9 | 9 | **0** | 0 | **0** | 0 | 2 | 2 | 2 | 2 | **3** | 3 | 4 | 4 | **6** | 8 | 8 | 9 | 0 | 1 | 2 | 5 | 6 | 6 | **7** | **7** |  |
|  |  | |  | 8 | **9** | **0** | 9 | 4 | **0** | 0 | **8** | 5 | 6 | 8 | 9 | 6 | **0** | 2 | 4 | 9 | **4** | 5 | **7** | 8 | 5 | 6 | 8 | 9 | **7** | 8 | 5 | 6 | **6** | 6 | 8 | 1 | 1 | 8 | 5 | 3 | 3 | 9 | **5** | **7** |  |
|  |  | |  | G | A | T | C | A | A | T | T | C | C | T | C | A | G | T | T | C | T | A | T | C | T | G | T | T | T | T | T | C | T | T | T | G | G | G | C | A | C | G | G | T |  |
| P | Guana | | LdH1 | . | . | . | . | . | . | . | . | . | . | . | . | . | **.** | . | . | . | **.** | . | . | . | . | . | . | . | . | . | . | . | . | . | . | . | . | . | . | . | . | . | . | **.** | 3 |
| i | cabibes | | LdH2 | . | . | . | . | . | . | . | . | . | . | . | . | . | **.** | . | . | . | **.** | . | . | . | . | . | . | . | . | . | . | . | . | . | . | . | . | . | . | G | . | . | . | **.** | 3 |
| n |  | | LdH3 | . | . | . | . | . | . | . | A | . | . | . | . | . | T | . | . | . | C | . | **C** | . | . | . | . | . | . | . | . | . | . | . | . | . | . | . | . | G | . | . | . | A | 1 |
| a |  | | LdH4 | . | . | . | . | . | . | . | A | . | . | . | . | . | T | . | . | . | C | . | **C** | . | . | . | . | . | . | . | . | . | . | . | . | . | . | . | . | G | . | . | . | A | 1 |
| r |  | | LdH5 | . | . | . | . | . | . | . | A | . | . | . | . | . | T | . | . | . | C | . | **C** | . | . | . | . | . | . | . | . | . | . | . | . | . | . | . | . | G | . | A | . | A | 1 |
|  | C | | LdH6 | . | . | . | . | . | . | . | A | . | . | . | . | . | T | . | . | . | C | . | **C** | . | . | . | . | . | . | . | . | . | . | . | . | . | . | . | . | G | . | . | . | A | 1 |
| d | a | | LdH7 | . | . | . | . | . | . | . | A | . | . | . | . | . | T | . | . | . | C | . | **C** | . | . | . | . | . | . | . | . | . | . | . | . | . | . | . | . | G | . | . | . | A | 1 |
| e | y | | LdH8 | . | . | . | . | . | . | . | A | . | . | . | . | . | T | . | . | . | C | . | **C** | . | . | . | . | . | . | . | . | . | . | . | . | . | . | . | . | G | . | . | . | A | 6 |
| l | u | | LdH8a | . | . | . | . | . | . | . | A | . | . | . | . | G | T | . | . | . | C | . | **C** | . | . | . | . | . | . | . | . | . | . | . | . | . | . | . | . | G | . | . | . | A | 2 |
|  | c | | LdH8b | . | . | . | . | . | . | . | A | T | . | . | . | . | T | . | . | . | C | . | **C** | . | . | . | . | . | . | . | . | . | . | . | . | . | . | . | . | G | . | . | . | A | 1 |
| R | o | | LdH9 | . | . | . | . | . | . | . | A | . | . | . | . | . | T | . | . | . | A | . | **C** | . | . | . | . | . | . | . | . | . | . | . | . | . | . | . | . | G | . | . | . | A | 1 |
| í |  | | LdH10 | . | . | . | . | . | . | . | A | . | T | . | . | . | T | . | . | . | C | . | **C** | . | . | . | . | . | . | . | . | . | . | . | . | . | . | . | . | G | . | . | . | A | 1 |
| o |  | | LdH11 | . | . | . | . | . | . | . | . | T | . | C | T | . | T | . | . | . | C | . | **C** | . | . | . | . | . | . | . | . | . | . | . | . | . | . | . | . | G | A | . | . | A | 1 |
|  | | | LdH12 | . | . | . | . | . | . | . | . | T | . | . | . | . | T | . | . | . | C | G | . | . | . | . | . | . | . | . | . | . | . | . | . | . | . | . | T | G | . | . | . | A | 1 |
|  | | | LdH13 | . | . | . | . | . | . | . | . | T | . | . | . | . | T | . | . | . | C | . | . | . | . | . | . | . | . | . | . | . | . | C | . | A | . | . | . | G | . | . | . | A | 1 |
| L | | | LdH14 | . | . | . | . | . | . | A | . | T | . | C | . | . | T | . | . | . | C | . | . | . | . | . | C | . | . | . | C | . | . | . | . | . | . | . | . | G | . | . | . | A | 1 |
| a | | | LdH15 | . | . | . | . | . | . | A | . | T | . | C | . | . | T | . | . | . | C | . | . | . | . | . | C | . | . | . | C | . | . | . | . | . | . | . | . | G | . | . | . | A | 5 |
|  | | | LdH16 | . | . | . | . | . | . | . | . | T | . | . | . | . | T | . | . | . | C | . | . | . | . | . | . | . | . | . | . | . | . | C | . | . | . | . | . | G | . | . | . | A | 6 |
| H | | | LdH17 | . | . | . | . | . | . | . | . | T | . | . | . | . | T | . | . | . | C | . | . | T | . | . | . | . | . | . | . | . | . | . | C | . | A | . | . | G | . | . | . | A | 1 |
| a | | | LdH18 | . | . | . | . | . | . | . | . | T | . | . | . | . | T | . | . | . | C | . | . | T | . | . | . | . | . | . | . | . | . | . | C | . | . | . | . | G | . | . | . | A | 3 |
| v | | | LdH18a | . | . | . | . | . | . | . | . | T | . | C | . | . | T | . | . | . | C | . | . | T | . | . | . | . | . | . | . | . | . | . | C | . | . | . | . | G | . | . | . | A | 1 |
| a | | | LdH18b | . | . | . | . | . | . | . | . | T | . | . | . | . | T | . | . | . | C | . | . | T | . | . | . | . | . | . | . | . | . | . | C | . | A | . | . | G | . | . | . | A | 1 |
| n | | | LdH19 | . | . | . | . | . | . | . | . | T | . | . | . | . | T | . | . | . | C | . | . | T | . | . | . | . | . | . | . | . | . | . | C | . | . | . | . | G | . | . | . | A | 1 |
| a | | | LdH20 | . | . | . | . | . | . | . | . | T | . | . | . | . | T | . | . | . | C | . | . | T | . | . | . | . | . | . | . | . | . | . | C | . | . | . | . | G | . | . | . | A | 1 |
|  | | | LdH21 | . | . | . | . | . | . | . | . | T | . | . | . | . | T | . | . | . | C | . | . | T | . | . | . | . | . | . | . | . | . | . | . | . | . | . | . | G | . | . | . | A | 3 |
|  | | | LdH21a | . | . | . | T | . | . | . | . | T | . | . | . | . | T | . | . | . | C | . | . | T | . | . | . | . | . | . | . | . | . | . | . | . | . | . | . | G | . | . | . | A | 1 |
|  | | | LdH22 | . | . | . | . | . | . | . | . | T | . | . | . | . | T | . | . | . | C | . | . | T | . | . | . | . | . | . | . | . | . | . | . | . | . | . | . | G | . | . | . | A | 1 |
|  | | | LdH23 | . | G | C | . | . | G | . | . | . | . | . | . | . | T | . | . | . | C | . | . | . | . | A | . | . | C | . | . | . | C | C | C | . | A | A | . | G | . | . | A | A | 1 |
|  | | | LdH24 | . | G | C | . | . | G | . | . | . | . | . | . | . | T | . | . | . | C | . | . | . | . | A | . | . | C | . | . | . | C | C | C | . | A | A | . | G | . | . | A | A | 1 |
|  | | A | LdH25 | . | **G** | **C** | . | . | **G** | . | . | T | . | . | . | . | T | . | . | . | C | . | . | . | C | . | C | . | **C** | A | . | . | **C** | C | . | . | A | A | . | G | . | . | **A** | A | 3 |
|  | | g | LdH26 | . | **G** | **C** | . | . | **G** | . | . | T | . | . | . | . | T | . | . | . | C | . | . | . | . | . | C | . | **C** | . | . | . | **C** | C | . | . | A | - | . | G | . | . | **A** | A | 3 |
|  | | r | LdH26a | A | **G** | **C** | . | . | **G** | . | . | T | . | . | . | . | T | . | . | . | C | . | . | . | . | . | C | . | **C** | . | . | . | **C** | C | . | . | A | - | . | G | . | . | **A** | A | 1 |
| M | | a | LdH27 | A | **G** | **C** | . | . | **G** | . | . | T | . | . | . | . | T | . | . | . | C | . | . | . | . | . | C | . | **C** | . | . | . | **C** | C | . | . | A | - | . | G | . | . | **A** | A | 2 |
| a | | m | LdH27a | A | **G** | **C** | . | . | **G** | . | . | T | . | . | . | . | T | . | . | . | C | . | . | . | . | . | C | . | **C** | . | . | . | **C** | C | C | . | A | - | . | G | . | . | **A** | A | 1 |
| t | | o | LdH28 | . | **G** | **C** | . | . | **G** | . | . | T | . | . | . | . | T | C | . | . | C | . | . | . | . | . | C | C | **C** | . | . | . | **C** | C | . | . | A | A | . | G | . | . | **A** | A | 1 |
| a | | n | LdH29 | . | **G** | **C** | . | . | **G** | . | . | T | . | . | . | . | T | . | . | . | C | . | . | . | . | . | C | . | **C** | . | . | . | **C** | C | . | . | A | A | . | G | . | . | **A** | A | 2 |
| n | | t | LdH30 | . | **G** | **C** | . | . | **G** | . | . | T | . | . | . | . | T | . | . | . | C | . | . | . | C | . | C | . | **C** | A | . | . | **C** | C | . | . | A | A | . | G | . | . | **A** | A | 1 |
| z | | e | LdH32a | . | **G** | **C** | . | . | **G** | . | . | T | . | . | . | . | T | . | . | T | C | . | . | . | . | . | C | . | **C** | . | . | . | **C** | C | C | . | A | - | . | G | . | . | **A** | A | 1 |
| a | |  | LdH32 | . | **G** | **C** | . | . | . | . | **C** | T | . | . | . | . | C | . | . | . | C | . | . | . | . | . | . | . | . | . | . | . | **C** | C | . | . | A | - | . | G | . | . | **A** | A | 3 |
| s | | B | LdH33 | . | **G** | **C** | . | . | . | . | **C** | T | . | . | . | . | T | . | . | . | C | . | . | . | . | . | . | . | . | . | . | . | **C** | C | C | . | A | A | . | G | . | . | **A** | A | 5 |
|  | | o | LdH33a | . | **G** | **C** | . | . | . | . | **C** | T | . | . | . | . | T | . | . | . | C | . | . | . | . | . | . | . | . | . | . | . | **C** | C | C | . | A | - | . | G | . | . | **A** | A | 4 |
|  | | l | LdH33b | . | **G** | **C** | . | C | . | . | **C** | T | . | . | . | . | T | . | . | . | C | . | . | . | . | . | . | . | . | . | . | . | **C** | C | C | . | A | A | . | G | . | . | **A** | A | 3 |
|  | | o | LdH33c | . | **G** | **C** | . | . | . | . | **C** | T | . | . | . | . | T | . | C | . | C | . | . | . | . | . | . | . | . | . | . | . | **C** | C | C | . | A | A | . | G | . | . | **A** | A | 1 |
|  | | n | LdH33d | . | **G** | **C** | . | . | . | . | **C** | T | . | . | . | . | T | . | C | . | C | . | . | . | . | . | . | . | . | . | . | T | **C** | C | C | . | A | A | . | G | . | . | **A** | A | 1 |
|  | | d | LdH33e | . | **G** | **C** | . | . | . | . | **C** | T | . | . | . | . | T | . | C | . | C | . | . | . | . | . | . | . | . | . | . | . | **C** | . | C | . | A | A | . | G | . | . | **A** | A | 1 |
|  | | r | LdH34 | . | **G** | **C** | . | . | . | . | **C** | T | . | . | . | . | T | . | C | . | C | . | . | . | . | . | . | . | . | . | . | . | **C** | . | C | . | A | A | . | G | . | . | **A** | A | 1 |
|  | | ó | LdH35 | . | **G** | **C** | . | . | . | . | **C** | T | . | . | . | . | T | . | C | . | C | . | . | . | . | . | . | . | . | . | . | . | **C** | . | C | . | A | A | . | G | . | . | **A** | A | 1 |
|  | | n | LdH36 | . | **G** | **C** | . | . | . | . | **C** | T | . | . | . | . | C | . | . | . | C | . | . | . | . | . | . | . | . | . | . | . | **C** | C | . | . | A | - | . | G | . | . | **A** | A | 1 |
|  | |  | LdH37 | . | **G** | **C** | . | . | . | . | **C** | T | . | . | . | . | T | . | . | . | C | . | . | . | . | . | . | . | . | . | . | . | **C** | C | C | . | A | A | . | G | . | . | **A** | A | 2 |
| S/V | | |  | S | **S** | **S** | S | V | **S** | S | **?** | S | S | S | S | S | **?** | S | S | S | **?** | S | **S** | S | S | S | S | S | **S** | V | S | S | **S** | S | S | S | S | S | S | S | V | S | **S** | **V** |  |

Sites from 1 to 810 correspond to *cytb* sequences; from 811 to 1217 correspond to NCR sequences.

Haplotypes with same number follow by a letter refer to haplotypes previously defined from *cytb* haplotypes (García-Machado *et al*. 2011). Identity with the first sequence is denoted by a dot. Sites that identify the three geographical groups of haplotypes defined in García-Machado *et al*. (2011) and those that identify discrete partitions within Pinar del Río and Matanzas haplotype groups are shaded and highlighted in bold. N represents the number of individuals per haplotype. S = transition, V= transversions, ? = both types of substitutions at the same site.
