# Supplementary material for: Go West: A One Way Stepping-Stone Dispersion Model for the Cavefish Lucifuga dentata in Western Cuba
Source: PLoS One. 2016 Apr 15;11(4):e0153545. doi: 10.1371/journal.pone.0153545 (PMC4833296; doi:10.1371/journal.pone.0153545)
Supplement: S3 Table — (DOCX) [file pone.0153545.s004.docx]

**S3 Table**

Mean pairwise genetic differentiation (*F_ST_*) estimates between the five geographic groups of populations.

| **Population group** | **Guanahacabibes** | **Cayuco** | **Havana** | **Bolondrón** | **Agramonte/ Jagüey Grande** |
| --- | --- | --- | --- | --- | --- |
| Guanahacabibes |  | * | * | * | * |
| Cayuco | 0.768 |  | * | * | * |
| Havana | 0.491 | 0.464 |  | * | * |
| Bolondrón | 0.912 | 0.883 | 0.652 |  | * |
| Agramonte/ Jagüey Grande | 0.888 | 0.87 | 0.642 | 0.682 |  |

* : Statistically significant (p<0.05) values.
